# A Federated Learning-Inspired Evolutionary Algorithm: Application to Glucose Prediction

**DOI:** 10.3390/s23062957

**Published:** 2023-03-08

**Authors:** Ivanoe De Falco, Antonio Della Cioppa, Tomas Koutny, Martin Ubl, Michal Krcma, Umberto Scafuri, Ernesto Tarantino

**Affiliations:** 1 ICAR-National Research Council of Italy, Via P. Castellino, 80131 Naples, Italy; 2 Natural Computation Lab, DIEM, University of Salerno, Via Giovanni Paolo II 132, 84084 Fisciano, Italy; 3 Department of Computer Science and Engineering, New Technologies for Information Society, University of West Bohemia, Technicka 18, 330 01 Pilsen, Czech Republic; 4 Department of Computer Science and Engineering, University of West Bohemia, Technicka 18, 330 01 Pilsen, Czech Republic; 5 Diabetology Center, First Department of Internal Medicine, University Hospital Pilsen, Alej Svobody 923/80, 323 00 Pilsen, Czech Republic

**Keywords:** federated learning, evolutionary algorithms, interpretable machine learning, diabetes

## Abstract

In this paper, we propose an innovative Federated Learning-inspired evolutionary framework. Its main novelty is that this is the first time that an Evolutionary Algorithm is employed on its own to directly perform Federated Learning activity. A further novelty resides in the fact that, differently from the other Federated Learning frameworks in the literature, ours can efficiently deal at the same time with two relevant issues in Machine Learning, i.e., data privacy and interpretability of the solutions. Our framework consists of a master/slave approach in which each slave contains local data, protecting sensible private data, and exploits an evolutionary algorithm to generate prediction models. The master shares through the slaves the locally learned models that emerge on each slave. Sharing these local models results in global models. Being that data privacy and interpretability are very significant in the medical domain, the algorithm is tested to forecast future glucose values for diabetic patients by exploiting a Grammatical Evolution algorithm. The effectiveness of this knowledge-sharing process is assessed experimentally by comparing the proposed framework with another where no exchange of local models occurs. The results show that the performance of the proposed approach is better and demonstrate the validity of its sharing process for the emergence of local models for personal diabetes management, usable as efficient global models. When further subjects not involved in the learning process are considered, the models discovered by our framework show higher generalization capability than those achieved without knowledge sharing: the improvement provided by knowledge sharing is equal to about 3.03% for precision, 1.56% for recall, 3.17% for $F_{1}$, and 1.56% for accuracy. Moreover, statistical analysis reveals the statistical superiority of model exchange with respect to the case of no exchange taking place.

## 1. Introduction

In Machine Learning (ML) [1], the problem of data privacy, i.e., the existence of data private to the owning subject, has become relevant in many application fields in these last years, as well shown in the recent survey in [2].

In ML, this data privacy issue was explicitly tackled for the first time in 2015 with the introduction of the concept of Federated Learning (FL) [3,4]; in the basic form of this approach, a server starts its execution by creating a random solution under the form of a model that is sent to a set of clients, one for each set of data that should be kept private. Learning on any local set of private data only takes place on the local client associated with those specific data, so it is never sent elsewhere. Learning on a node results in the model being modified locally to best adhere to the local data. The locally modified model is sent back to the server at the end of the local learning. Once the latter has received all the modified models, it aggregates them to create a new model that considers all the local learning. After the aggregation phase, this latter model is sent again to the clients, and the process continues until it reaches a termination criterion on the server. A good survey on recent advances in FL can be found in [5].

Not all the existing ML techniques can be used within FL, because only some can undergo a meaningful aggregation process. For example, there is no such aggregation process available for methods based on Deep Neural Networks (DNNs) [6], logistic regression [7] and Radial Basis Functions [8].

Another important issue relevant to ML is the interpretability of the proposed solutions, as well evidenced in [9]. This means that a proposed solution should be understandable by any user. This is clearly a problem with the recent and numerically well-performing ML techniques, which DNNs [10,11] are. The latter build an internal model that, although capable of excellent numerical performance, is unintelligible to the user, be it a physician or a patient, which is why they are called black boxes.

To somehow get rid of this problem, in 2017, Explainable Artificial Intelligence (XAI) (https://sites.google.com/view/fl-tutorial/?pli=1 accessed on 8 February 2023) [12] was introduced, which tries to endow DNNs with mechanisms allowing the creation of an external model that can somehow explain the behavior of the algorithm in making its decisions [13]. The problem with this is that there cannot be any guarantee that the external model is the same as the internal one over the data domain, which could lead to serious errors that could even be fatal in the medical domain.

This paper proposes a general ML framework that can satisfy the issues related to data privacy and interpretability. Namely, our approach is based on the use of Evolutionary Algorithms (EAs), a widely used class of ML methodologies [14,15,16], and consists of the use of a distributed version of an EA (dEA) [17,18].

The proposed methodology is close to the classical FL approach, yet it is simultaneously different from it. It is similar because it allows each client to work on local data only and because global knowledge of the problem is obtained by aggregating the different local knowledge. The difference with the classical FL is that this aggregation is performed implicitly rather than explicitly, as in the typical FL scheme. A thorough explanation of this difference is given later in the paper.

From an architectural viewpoint, the dEA framework we put forward contains both a master acting as the server and, thus, managing the algorithm, and a set of nodes, each of which represents a client and only contains local data to be kept private. Grammatical Evolution (GE) [19,20] is used as the specific EA: in it, each proposed solution is a model constituted by an expression linking (some of) the problem variables, so it represents explicit knowledge that users can immediately understand. Given this choice, we make use of a distributed GE scheme.

In our proposed framework, learning takes place locally on each node, on which good local knowledge specific to the local data is gained in terms of a local model. Moreover, at given times, these good local models are sent to the master. This latter evaluates the global quality of each of these models over all the data, thanks to the help of all the nodes, without any need to transmit data from any node; then, it sends to all of the nodes all these models. These arrived solutions enter the local learning process; in this way, the local knowledge in each node can be augmented thanks to that arriving from other nodes. As a consequence of this exchange of information, better global knowledge can be obtained.

This kind of implicit aggregation process ties local models into a global one. Said otherwise, during the execution of our algorithm, global knowledge emerges from the data contained in the various local sets without any need to physically exchange or make them visible. At the end of the execution, the model performing the best globally, i.e., over all the local sets of private data, is obtained as desired. Moreover, as a very interesting byproduct of our framework, apart from the global knowledge of the problem, for each set of private data, personalized knowledge is obtained that is specific to each of them.

The proposed framework does not deal with the security issue in the current implementation. For a thorough description of the problems of privacy guarantees for users and detection against possible attacks, interested readers can refer to [21,22].

The framework is applicable in different domains [23,24]: healthcare, bank loans, advertising, financial fraud, and insurance, among others. In this paper, we focus our attention on the medical field, where there is a high need for data privacy and interpretability of the solutions.

Regarding privacy, medical data are highly sensitive and strictly personal to the patient, so they should not be disclosed to anybody else, meaning both any other patient participating in the study and any person involved in the handling of the data or the learning process. In the European Union, this issue is regulated by the General Data Protection Regulation (GDPR) (2016/679 law) (https://eur-lex.europa.eu/eli/reg/2016/679/oj accessed on 3 February 2023) [25] that concerns data protection and privacy within the Union.

For interpretability, in medicine, a solution should be understandable by any subject participating in the study. This holds for a physician wishing to evaluate from a medical viewpoint the soundness and the interest of the knowledge proposed by the ML system, or a patient wishing to receive a diagnosis that is clear and well explains the reasons for that decision. This also follows the GDPR law, specifically Article 12, which states that the data controller gives information to the ‘data subject in a concise, transparent, intelligible and easily accessible form, using clear and plain language’. Moreover, Article 25 recognizes subjects’ right to contest any automated decision making that was solely algorithmic.

Within the medical field, we chose to take into account diabetes [26] disease, with specific reference to the prediction of future glucose values for subjects suffering from Type-1 diabetes mellitus (T1DM). Diabetes is a chronic disease, and its T1DM version is characterized by the fact that the subject’s pancreas produces practically no insulin, which calls for a life-lasting treatment consisting in the daily administration of amounts of insulin. In fact, if not treated, diabetes determines hyperglycemia, a condition of increased blood glucose values that with time may yield relevant damage to several parts of the body, among which are the eyes, kidneys, nerves, heart, lower limbs, and blood vessels [27].

As the data set to conduct our experiments, we avail ourselves of the well-known and publicly available Ohio T1DM data set [28]. In the experiments, rather than attempting to predict the exact future glucose values, as  would be the case in multivariable regression, we treat prediction as a classification problem. This is an approach already taken in the scientific literature through various methods [29]. To follow this way of operating, we divide the glucose range into seven intervals, and for each future value, we aim at predicting the interval it lies within. This is a good way to predict if a future glucose value will lie in high-risk intervals, such as those associated with very low or very high values. In this case, immediate recovery actions can be taken to eliminate or reduce risks to the subject’s health.

In clinical practice, Time-in-Range represents the time spent within a safe glucose-level range [30]. Within the safe range, the patient may avoid unnecessary actions to correct the blood glucose level, which may accidentally trigger an undesired outcome. In principle, the patient needs to know whether he/she is staying within the safe range or deviating from it. The proposed prediction addresses this need, while relieving the patient from the stress of operating with exact glucose levels, which may lead to diabetes burnout [31]. As a side effect, the resulting models can be simpler, thus reducing the computational complexity for lower-power devices and for possible cloud processing for thousands of patients. Moreover, the proposed method has the future potential to be applied as a watchdog over an insulin pump’s controller activity. As it is a prediction method, it could detect the controller’s failure to keep glucose levels in the safe range ahead of time.

As the outcome of our experiments, we expect to obtain an explicit global model able to perform generalization. This means that such a model should perform acceptably well on all the subjects involved in this learning process and on others not involved in creating the model. This would be highly important in real-world situations where we have to start monitoring diabetic subjects for which we do not have specific knowledge. We would need a general model to use on them to predict their future glucose values, and we could use the one obtained through our framework.

To evaluate the effectiveness of the mechanism of information exchange among nodes, our algorithm is experimentally compared against a distributed EA differing only in the absence of exchange. This comparison is effected both in terms of numerical performance achieved in the classification and from the statistical analysis perspective.

The rest of this paper is structured as follows. Section 2 presents a brief state-of-the-art review. Section 3 describes the proposed collaborative approach and the data set used. The experimental framework and the discussion about findings are reported in Section 4. In the same section, the results of the statistical analysis test, performed over the twelve subjects of the complete Ohio T1DM data set, are outlined. The last section exposes the conclusions and provides some indications on future work.

## 2. State of the Art

An important issue when dealing with ML applications is data privacy related to the protection of sensible personal information. This issue is increasing with the usage of online platforms collecting private data to provide services. A privacy-preservation framework must ensure high protection to let individuals share their information. FL represents the most employed technology to accomplish the privacy task [3,4,32]. This federated technique facilitates distributed collaborative learning by multiple clients under the coordination of a server. Data privacy is assured by training a prediction model through decentralized data, locally associated with different clients and not exchanged or transferred. Federated Learning is applied to support privacy-sensitive applications in several fields [24].

Another important issue of ML lies in its ability to discover underlying explanatory structures. The most performing techniques, i.e., deep learning neural networks, can be regarded as black boxes lacking an explicit knowledge representation. Utilizing black box learning models involves difficulty in understanding what model inputs drive the decisions (explainability) and, above all, prevents specialists from understanding the reason for a prediction (interpretability) [9,33]. The demand for transparent decisions pushes towards explainable and interpretable systems [34]. Explainable systems are black box learning models endowed with external XAI tools, without guarantee that these external tools allow capturing the internal model behavior. Interpretable models are models able to explain themselves by providing explicit models. From now on, the term interpretability is employed with the above meaning.

Both the above issues assume noticeable importance in the medical domain, e.g., diabetes management. Several techniques have been investigated to discover data-driven glucose forecasting models, ranging from approaches based on regression [35,36,37,38,39] to those that handle the prediction as a classification problem [29,40,41,42]. These techniques can be classified as explainable or interpretable based on the techniques employed for discovering the learning model.

Leaving aside the regression-based models, a brief literature survey on the state-of-the-art works on diabetes classification using data-driven ML models is conducted for the explainable and interpretable models described above. The review is related to recent articles that explore different techniques for dealing with glucose prediction formulated as a classification problem.

The first category concerns explainable techniques, most based on neural models, that exhibit outstanding performance at the expense of the difficulty of comprehending the aspects that can explain the decision, even when enriched with external XAI explanation tools [43,44,45,46,47,48,49,50,51,52,53,54,55]. As already illustrated in the introduction, the lack of explanation could yield the usage of these classification techniques problematic in the medical domain [56]. In fact, these learning models’ inner workings are too complicated to understand for physicians.

The second category includes interpretable models characterized by explicit prediction models. Most of these models rely on decision trees [57,58,59,60,61,62,63,64,65,66,67,68,69]. Although the methods based on these trees [70] could provide explicit knowledge, in many cases, it is challenging to linearize the resulting acyclic decision graphs into simple decision rules. Other attempts have been carried out to make predictions through classification rules based on if–then–else conditions induced by an evolutionary approach [71,72].

Independently of the belonging category, none of the above-examined approaches consider the problem of data privacy, which remains a critical concern when handling sensitive information such as diabetic data [73]. FL technology has been utilized in the medical domain to train a prediction model through decentralized data for dealing with different problems [74,75,76,77].

Only some recent papers contemplate the problem of implementing privacy-protected diabetes prediction systems relying on FL approaches and encryption with different training processes [78,79]. However, instead of employing data related to a single patient, the training concerns data collected in each hospital [78] or grouped by defining cohorts associated with diabetes-related complications [79]. Therefore, while ensuring data protection, these approaches do not permit the development of personalized models that are important from the point of view of precision medicine.

This review makes us confident that, at least in the recent scientific literature, data privacy has not been considered for tuning interpretable glucose forecasting models for diabetic patients. Indeed, most reviewed predictive models rely on centralized training data, or refer to decentralized training clients associated with data not referred to single patients, and thus are unable to allow personal disease treatment and prevention strategies. Table 1 summarizes the results of the review. As evinced from this table, the limitation of the current FL approaches is related to solution interpretability.

We aim to overcome this limitation by dealing with a data-privacy paradigm able to discover interpretable Machine Learning models for glucose prediction. This paradigm is based on some collaborative concepts inspired by FL. Specifically, this collaboration is pursued by training a Federated Learning-inspired global model, relying on a dEA that evolves multiple decentralized clients, each representing a single patient, holding local data samples without exchanging them. The collaborative training consists in sharing the model discovered by each local patient to be aggregated in a global model. The personalized models emerged at the end of evolution can be exploited for personal diabetes treatment or aggregated and used as global models.

Regarding interpretability, we concentrated on a grammar-based Evolutionary Algorithm to discover explicit classification models.

The following section illustrates the devised framework and its specific application to the glucose forecasting problem.

## 3. Methods and Materials

### 3.1. The Proposed Approach

This paper introduces a novel Federated Learning-inspired Evolutionary Algorithm (FLEA). The proposed methodology is a master/slave dEA [18,80] in which each slave runs a canonical sequential EA, and individuals, i.e., predictive models, can synchronously migrate between populations with a given frequency [81]. Specifically, each slave evolves a population of predictive models using learning data from a specific slave exclusively. In particular, the proposed method works as follows. During the evolution:

- at specific instants of time (migration interval), the best model evolved so far on each slave is sent to the master node;
- the master node returns the collected models to all the slaves, which evaluate them on the local data, thus preserving data privacy;
- each slave uses the immigrant models within its population by replacing as many local individuals as possible with the lowest performance if better. In this way, the evolved models on each slave node receive information about the evolving models on the other slave nodes. Thanks to the mechanisms of selection, replacement, and genetic variation, the slave nodes can integrate the incoming information into their own population.

**Figure 1:**
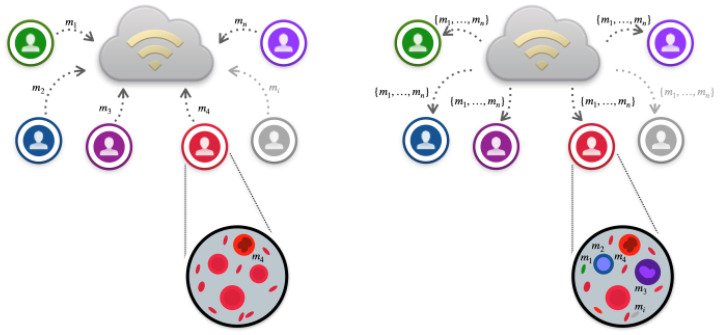
FLEA migration. The left side of the figure sketches the migration from the slaves to the master, while the right one traces the migration from the master to the slaves. The largest circles show single slaves with the corresponding local individuals, i.e., prediction models ($m_{1},\ldots ,m_{n}$). In particular, the individuals internal to the left-side circle have the same color because the models are all related only to local data. Differently, the individuals inside the right-side circle indicate that each slave, after communication with the master, can integrate the information from the immigrants by exploiting the mechanism of selection, replacement, and genetic operators, as evidenced by the color overlapping of the single individuals.

The above steps are graphically outlined in Figure 1.

At the end of evolution, each slave sends the best predictive model found to the master node that collects all of them. Then, the master node sends to each slave the list of all the best local models just received. Each slave evaluates such models on its local data and sends the list of their performance to the master node. This last step is particularly important when the proposed method is inserted in a system that continuously optimizes models on local data. In fact, when new local data are added to the system, the master node could provide the initial population of a new slave node with the predictive models coming from the other individuals, thus allowing a boost in the search for the specific local model on these new data.

Algorithms 1 and 2 report the pseudocode for the master and slave, respectively. It is worth noting that the proposed methodology is very close to an FL approach [5]. The differences lie in the fact that, in the federated approach, the integration of patterns is explicitly performed by the master node, and the communication of learned patterns is direct. In contrast, in the proposed approach, the integration of patterns is implicitly performed by the slave nodes, in that it is demanded of the mechanisms of selection and genetic variation, i. e., crossover [82], that, eventually, perform the integration. The communication is indirectly effected through the master node.

### 3.2. The Data Set

The FLEA framework is investigated to forecast future glycemic trends for T1DM patients. The experiments are conducted on the Ohio T1DM data set, released in 2020 [28], which gathers data of T1DM patients. This data set was collected at the Ohio University and contains data related to twelve subjects, each of whom was monitored with a Continuous Glucose Monitoring (CGM) system for about eight weeks while being on insulin pump therapy. Given the availability of data in the dataset related to, among others, measured subcutaneous glucose, injected insulin (basal plus boluses), and carbohydrate ingested during the day (time and estimated size of all meals), future glucose values can be predicted on the basis of the sets of current and recent values available for these three parameters. The sampling interval of glucose measurements achieved by the CGM system is equal to Δt=5 min. Each slave only contains the private data associated with a single patient.

To allow supervised learning, the data series of each patient are partitioned into training and testing sets, respectively, used to extract the model during the learning phase and assess its quality over unseen samples. The supervised learning phase is carried out on the six patients related to the data set released in 2018 [83], while a successive validation phase is performed on the testing sets of the remaining six patients added to the data set in 2020. The information about the number of training and testing samples for each patient is reported in [28].
**Algorithm 1** Pseudocode of FLEA on the master nodeset global stopping condition to FALSE**while** not global stopping condition **do**    **for** each slave **do**        receive the best local model    **end for**    **for** each slave **do**        send the list of the received best local models    **end for**    **for** each slave **do**        receive the local stopping condition    **end for**    **if** all local stopping conditions are TRUE **then**        set global stopping condition to TRUE    **end if****end while****for** each slave **do**    receive the final best local model**end for****for** each slave **do**    send the list of the final best local models    receive the list of the fitness of the final best local models**end for**

**Algorithm 2** Pseudocode of FLEA on a slave node
randomly generate an initial population of *n* modelsevaluate the fitness of each modelset the maximum number of generations $g_{\max}$set current generation *g* equal to 0set local stop condition to FALSE**while** $g<g_{\max}$ **do**    **if** migration time is TRUE **then**        send the best local model to the master node        receive from the master node the best local models of the other slaves (immigrants)        evaluate on the local data the best local models received        replace the worst local models with the immigrants if better    **end if**    generate the new population according to the chosen EA    evaluate the fitness of the models in the new population    **if** some local conditions hold **then**        set local stop condition to TRUE    **end if**    send to the master node the local stop condition    g=g+1
**end while**
send the final best local model to the master nodereceive from the master node the list of final best local models of the other slavesevaluate the fitness of each final best local model on local datasend to the master node the list of the evaluated fitness of the final best local models


#### Data Preprocessing

As regards the data preprocessing, we performed the following arrangement:

- Samples with missing glucose readings in training and testing sets are thrown away to avoid that the predicting model can be the result of artificial observations;
- Insulin and carbohydrates data were aligned to the closest CGM glucose reading time;
- No outlier detection and no data normalization were effected.

It is pointed out that the discrete signals of administered insulin, i. e., insulin boluses plus insulin basal, and the assumed carbohydrates are to convert into continuous signals to estimate their impact on the glucose values over time. The Hovorka model [84], simulating the absorption rate of the injected insulin through a two-compartment chain, is employed for preprocessing the injected insulin boluses. This model permits adding the signal delineating the absorption rate of the boluses to the signal representing the absorption rate of subcutaneously administered long-acting insulin.

Let us assume that the glucose level G(t), the injected insulin U(t), and the consumed carbohydrates $D_{g}\left(t\right)$ are available. The model for insulin absorption is

(1) $$\begin{array}{cc} \frac{dS1}{dt} & =U\left(t\right)-\frac{S1}{t_{\max_{I}}} \end{array}$$

(2) $$\begin{array}{cc} \frac{dS2}{dt} & =\frac{S1-S2}{t_{\max_{I}}} \end{array}$$

in which S1 and S2 are the two compartments making up the chain for modeling the absorption of subcutaneously infused short-acting insulin, U(t) [mU min${}^{-1}$] is the amount of injected insulin, $t_{\max_{I}}=55$ [min] is the constant indicating the time-to-maximum insulin absorption, and S1(t) [mU] and S2(t) [mU] are the amounts of insulin in the two compartments. Then, the plasma insulin concentration *I* [mU L${}^{-1}$] is described as

(3) $$\begin{array}{cc} \frac{dI}{dt} & =\frac{S2}{V_{I}\cdot t_{\max_{I}}}-k_{e}\cdot I \end{array}$$

where $k_{e}=0.138$ [min${}^{-1}$] is the fractional elimination rate of the insulin from plasma and $V_{I}=0.12$ [L kg${}^{-1}$] is the insulin distribution volume. The constant values are derived from Hovorka’s model [85].

Regarding the carbohydrate intake, in the presence of a meal, the gut absorption rate is modeled in accord with [84] as

(4) $$C\left(t\right)=\frac{D_{g}\cdot A_{g}\cdot t\cdot e^{-t/t_{\max}}}{t_{\max}^{2}}$$

where $t_{\max}=40$ [min] is the time-of-maximum appearance rate of glucose in the accessible compartment, $D_{g}$ is the amount of digested carbohydrates, and $A_{g}=0.8$ is the carbohydrates bioavailability [86]. This function rapidly increases after the meal and then lowers to 0 in 2–3 h. Outside such a period, the values of missing carbohydrate are filled with zeroes.

At the end of the preprocessing, by integrating Equation (3) and exploiting Equation (4), we have two signals, discretized every Δt minutes, for the absorbed insulin and carbohydrates, i.e., I(t) and C(t), respectively. More specifically, when at time *t* there is an insulin release or carbohydrate intake event, their absorbed quantities are propagated over time through Equations (3) and (4) from the current time *t* ahead and, if needed, summed to the residual quantity evaluated by the compartment model in the past. Typically, the variation range of G(t) is about $\left[2÷25][\mathrm{mmol}\;L{}^{-1}\right]$, of I(t) is about $\left[0÷10][\mathrm{mU}\;L{}^{-1}\right]$, and of C(t) is [0÷3][g].

### 3.3. FLEA to Forecast Future Glycemic Trends

Forecasting future glycemic trends for T1DM patients can be regarded as a multivariate time series regression problem, falling within the learning of data-driven models exploiting information extracted from CGM systems. To apply the FLEA framework to the above problem, we exploit the capability of the Grammatical Evolution [82] to automatically evolve interpretable regression models.

Moreover, differently from other EAs, GE explicitly makes use of the context-free grammars that are able to design a specific form for the evolved models.

To do this, we need to define a suitable grammar and a fitness function. Moreover, rather than attempting to predict the exact future glucose values, we transform the time series regression into a classification problem.

#### 3.3.1. The Grammar

**Figure 2:**
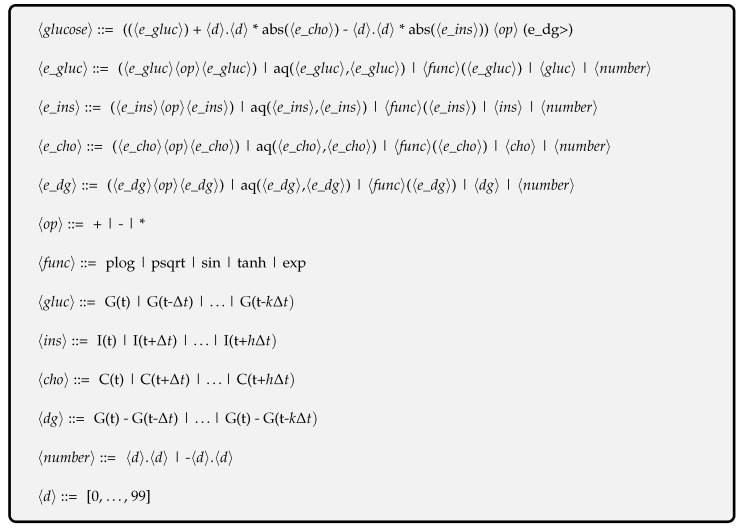
The grammar for the glucose forecasting model (Equation (5)).

The context-free grammar in Figure 2 depicts the syntax of the GE-based expressions evolved on each slave, where 〈gluc〉 represents the glucose levels in the past, 〈ins〉 and 〈cho〉 indicate insulin and carbohydrates to be absorbed in the future, and 〈dg〉 is the difference between the current and past glucose levels, respectively. In our grammar, the protected psqrt and plog functions return the square root of the absolute value of the argument, the logarithm of the summation of 1, and the absolute value of the argument, respectively, while aq stands for the protected analytic quotient operator [87]. Table 2 outlines the protected functions utilized in the grammar.

By considering the values of G(t) every Δt minutes in a time window of kΔt minutes before the current instant *t*, as well as the values of I(t) and C(t) every Δt minutes in a time window of hΔt minutes after the current instant *t*, we search for an explicit regression model to predict the future glucose value $\hat{G}\left(t+h\Delta t\right)$ at a forecasting horizon hΔt:

(5) $$\begin{array}{c} \hat{G}\left(t+h\Delta t\right)=(\Gamma \left(G(t),G(t-\Delta t),\ldots ,G(t-k\Delta t)\right)-\Theta (I\left(t\right),I\left(t+\Delta t\right),\ldots ,I\left(t+h\Delta t\right))\\ +\Omega \left(C(t),C(t+\Delta t),\ldots ,C(t+h\Delta t)\right))\;◊\;\Phi \left(dG(t,t-\Delta t),\ldots ,dG(t,t-k\Delta t)\right) \end{array}$$

where the symbol ◊ represents an algebraic operation in the set: {+,−,·}, and Γ,Θ, Ω, and Φ are expressions on *G*, *I*, *C*, and dG, respectively.

#### 3.3.2. From Regression to Classification

Glucose prediction is typically performed through multiseries regression to predict glucose values as accurately as possible. Nonetheless, in the literature, the use of classification to forecast glucose ranges rather than exact values is becoming more and more popular [47,48,49,88]. This is of great help when high-risk situations such as hyperglycemic events or, even more crucially, hypoglycemic ones should be forecasted with good advance. In these cases, it is more important to predict the occurrence of such an event than the precise glucose values.

To perform classification, the continuous glucose values were mapped into seven intervals, leading to a seven-class problem. More precisely, two classes make reference to hypoglycemia, three relate to euglycemia (normal values), and two refer to hyperglycemia.

The decision to consider two hypo- and two hyperclasses is based on the outcome of the international consensus held in 2017 and reported in [89]. Following that consensus, we used the same bounds as in that document; the corresponding bounds are reported in Table 3.

As concerns the euglycemic range, unlike [89], we prefer instead to consider a division into three classes, as reported in Table 3. The rationale for this is that if we had just one normal/target range, then we would not be able to track possibly dangerous, out-of-target-range deviating glucose development: we would directly pass from a series of normal values to the occurrence of a hypoglycemic event, without any warning. Instead, by using three classes, the middle one being larger and the two border zones towards hypo- and hyper-being ‘thin’, we would obtain warnings before a hypo- or a hyperglycemic event took place. In fact, for hypoglycemia, we would have a series of normal values, followed by a (series of) normal-closing-to-hypovalues, followed by hypovalues.

In Table 3, for each class, the ID we assigned to it is displayed, the corresponding glucose value range is shown both in mmol/L and in mg/dL, and the action(s) required during the monitoring are reported.

In this way, the problem is transformed into a classification task, and the aim is to predict the class of any glucose value in the future, starting from the available values for glucose, absorbed insulin, and carbohydrates, as expressed in Equation (5).

**Figure 3:**
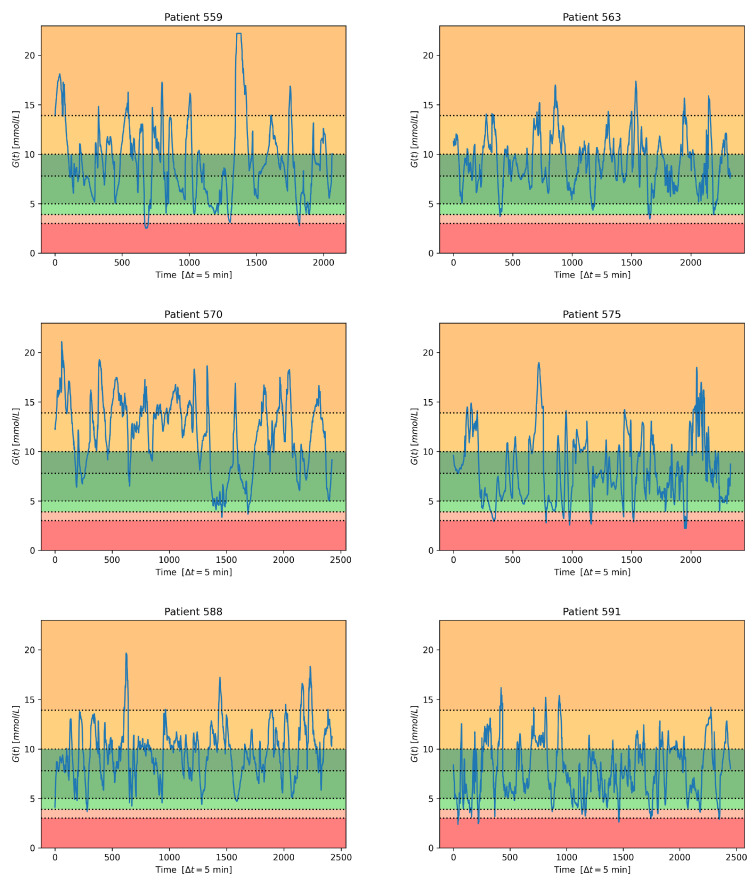
Classification bands on the testing set for each patient in the Ohio data set [83].

Figure 3 shows, for the testing set of each subject, the transformation of the continuous glucose signal into the corresponding set of items for the seven-class classification task considered in this paper.

Table 4 reports the number of samples in the training (Tr) and testing (Ts) sets for each patient and class. From the table, it can be easily seen that, for all six subjects, the three classes related to normal values and the two for the hyperglycemic values are much more populated than the two corresponding to ’very low’ and ’low’ glucose values. The latter two often only contain few values, and it is worth noting that for subjects 563, 570, and 588, the ’very low’ class is even empty. This means that all the six data sets are highly unbalanced, which is a complication in classification [90].

#### 3.3.3. Fitness Function

To evaluate the quality of any solution proposed, a suitable fitness function should make reference to the specific metrics typically used for this kind of problem, as, e.g., accuracy, sensitivity, specificity, area under the ROC curve, $F_{1}$ score, Matthews correlation coefficient, and so on.

We decided to use the *F*_1_ score, since the data sets corresponding to each of the six subjects investigated here are highly unbalanced; especially, their classes 0 (very low glucose values), 1 (low glucose values), and, for some subjects, 6 (very high glucose values) contain very few items with respect to the other four classes.

It is well known that, whenever a data set is highly unbalanced in terms of number of items contained in the different classes, as it is the case here, metrics such as accuracy, sensitivity, and specificity are not suitable: good performance on the most populated class(es) could lead to numerically good results without actual learning taking place on the least populated class(es). This could mean that every time an item belonging to one of the minority classes has to be classified, the algorithm could wrongly assign it to one of the majority class(es).

For unbalanced data sets, instead, metrics such as $F_{1}$ score or Matthews correlation coefficient can more effectively take this problem into account.

For a two-class problem in which we have a positive class and a negative one, $F_{1}$ score is computed as

(6) $$F_{1}=\frac{tp}{tp+0.5\cdot (fp+fn)}$$

where:

- *tp*: the number of true positives, i.e., the items in the positive class that are correctly assigned to that class;
- *fp*: the number of false positives, i.e., the items in the negative class that are incorrectly assigned to the positive class;
- *fn*: the number of false negatives, i.e., the items in the positive class that are incorrectly assigned to the negative class.

When, instead, there are more than two classes, as in this case, the definition of $F_{1}$ score can be generalized in several ways. Within this paper, we used the method of weighted averaging, This means that the resulting $F_{1}$ score value accounts for the contribution of the $F_{1}$ score computed for each class and weighted by the number of items of that given class. In formula:

(7) $$F_{1}=\frac{\sum_{n=1}^{nc}p_{i}\cdot F_{1i}}{nc}$$

where *nc* is the number of classes in the data set, $p_{i}$ is the percentage of items in the *i*-th class, and $F_{1i}$ is the $F_{1}$ score value computed on the *i*-th class.

The admissible range for $F_{1}$ score is [0.0–1.0], and higher values represent better classifications. By choosing this metric, the classification problem becomes a maximization one.

## 4. Experimental Results

### 4.1. Experimental Framework Setting

Our approach was implemented by exploiting PonyGE2, a freely downloadable and patent-free GE implementation in Python [82]. PonyGE2 has a number of GE-specific parameters to set, the meaning of which can be found in [82]. After a preliminary tuning, the parameters used for all the experiments were set as follows: population size and maximum generations equal to 200 and 500, respectively; codon size equal to 100,000, tournament selection with size 4, mutation probability equal to 10%, one-point crossover probability equal to 90%, int flip per codon mutation with one mutation event, and Position Independent Grow method for the individual initialization. For the slaves, a single local stopping conditionwas considered and set to the fulfillment of the maximum number of generations.

We set the communication between the master and the slaves to take place every 100 generations. This value was chosen because of two motivations.

The first reason comes from the field of dEAs: it is known that any subpopulation should not receive immigrating individuals too frequently, because this would perturb the local evolution at each communication time. The local search must be given sufficient time to suitably integrate the arrived individuals into the local subpopulation, so as to exploit their good features.

The second reason is related to the FL principles themselves in terms of security: an FL algorithm should involve the least possible amount of information being transmitted, because any possible communication could be attacked, possibly resulting in a subject’s relevant information being disclosed or in an injection of fake data by attackers, which could lead to totally wrong learning.

Hence, based on our experience, we feel the value of 100 implies the lowest amount of communication that allows improvement in the learning process while, at the same time, not excessively exposing the process to external attacks. In fact, this value of 100, together with the number of generations being set to 500, means that, during the whole execution, only five communication phases between the master and the slaves take place.

The forecasting horizon is hΔ(t)=30 min, because the forecasting accuracy becomes worse and less reliable as the prediction horizon augments [91,92]. A horizon longer than 30 min, e.g., 2 or 4 h, is only practical for time spans that refer to almost steady-state situations as nocturnal predictions when sleeping. Any external event can cause a substantial and unpredictable glucose-level variation during these long intervals. The considered past time window is kΔ(t)=60 min for the historical samples leveraged for the forecasting. The time span for the historical data is chosen considering that 30-min data in the past are enough to perform an effective prediction [93]. Given that the values are taken at 5-min intervals, the values for *k* and *h* are equal to 12 and 6, respectively. This implies that both in the grammar and in Equation (5), at each time t, we consider twelve glucose values in the past and six insulin and carbohydrate values in the future.

To assess the effectiveness of the proposed approach, we conducted two experiments:

- In the first experiment, we used a non-FL approach consisting of FLEA with no communication between the master and the slave nodes during the evolution. In other words, we executed a separate optimization for all the patients, thus obtaining for each of them a personalized model. At the end of the executions, we collected all the models and selected among all of them the model with the best average performance on all the patients;
- In the second experiment, we used FLEA. The average outcomes for each run were evaluated at the end of the evolution by considering all the best local models received by the master node from all the slaves and measuring their performance on all the patients to evaluate how they perform on average when adopted as global models.

For each patient, indicated with the identifier ID, twenty runs were carried out to reduce the randomness in the GE algorithm initialization. The evaluation is performed on all instances for which a glucose measurement is available over the prediction period.

### 4.2. Findings and Discussion

Table 5 and Table 6 report the $F_{1}$ score of the best models on each slave of both experiments, and the last row and column show their averages and standard deviations. By looking at Table 5, it can be evidenced that, when adopted as a global model, each personalized model exhibits $F_{1}$ score values that are quite different on a specific patient (rows), and the same is true also when the performance of the models is measured on a specific patient (columns). On the contrary, inspecting the results of Table 6 related to our approach, the scenario changes. Independently of the adopted global model, the average $F_{1}$ score of the evolved models is always better than the previous case, except for subject 570, and very close to each other (rows). A similar consideration also holds for all the models on a specific patient (columns), thus evidencing that the proposed approach can evolve generalized models exhibiting better performance. Moreover, if we look at the best models evolved on local data (diagonals in the tables), it is evident that communication helps improve the performance on local nodes too.

**Figure 4:**
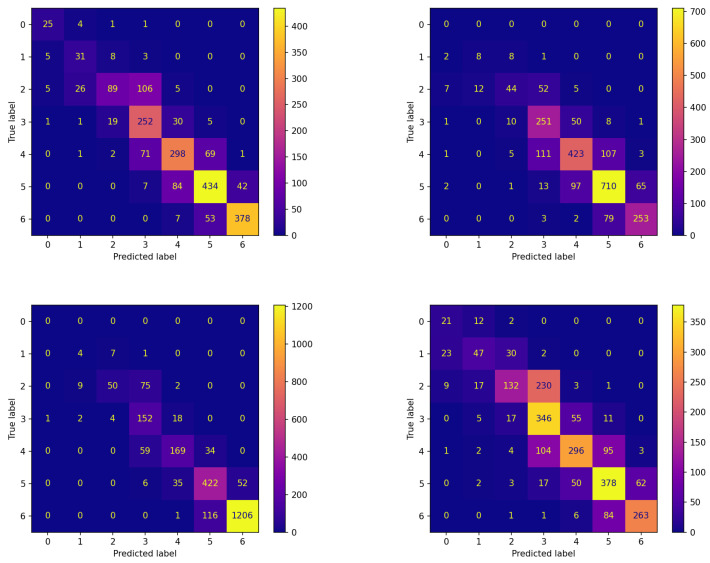

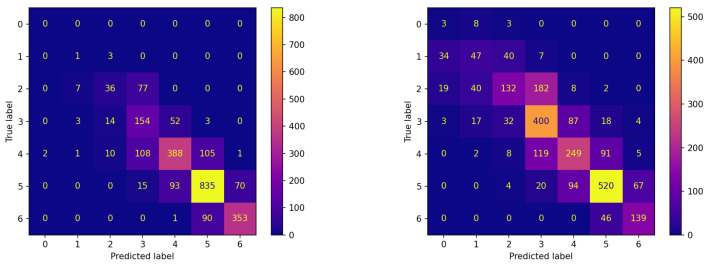
Confusion matrices on the testing set of the best model (model 570) evolved by the non-FL approach on each patient.

**Figure 5:**
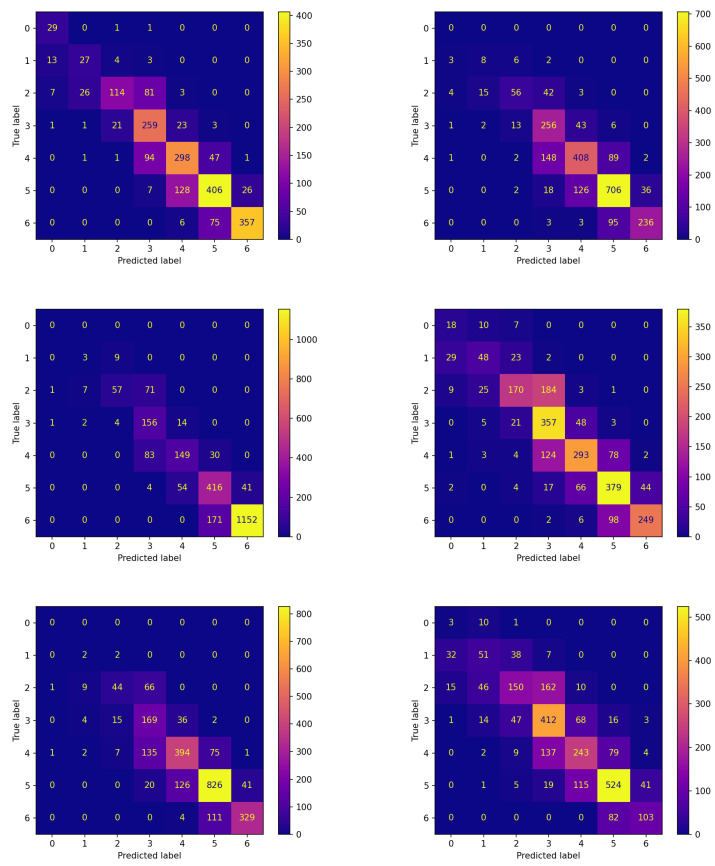
Confusion matrices on the testing set of each patient for the best model (model 575) evolved by FLEA algorithm.

Analogous reflections can be made by comparing the corresponding panes of Figure 4 and Figure 5 reporting the confusion matrices on the testing set for both experiments. This comparison documents that FLEA frequently increases the number of items correctly assigned to the classes. This can be verified by looking at the cells along the diagonals, which in most cases contain higher values.

Once we found the two global models proposed by the two different approaches, we wished to investigate their generalization capability to ascertain whether or not they have similar performance for this issue. To this aim, we executed them on six more patients in the Ohio data set. Table 7 reports the corresponding results in terms of $F_{1}$ score for the two models over the testing sets of these six additional patients. The last column in the table reports the average and the standard deviation of these $F_{1}$ values.

The table reports that, over all six subjects, the model obtained by FLEA always performs better than that achieved by the non-FL approach. This is very important, because it allows concluding that the model provided by our FLEA framework is more general; therefore, it can be used for new subjects not participating in the learning process.

**Figure 6:**
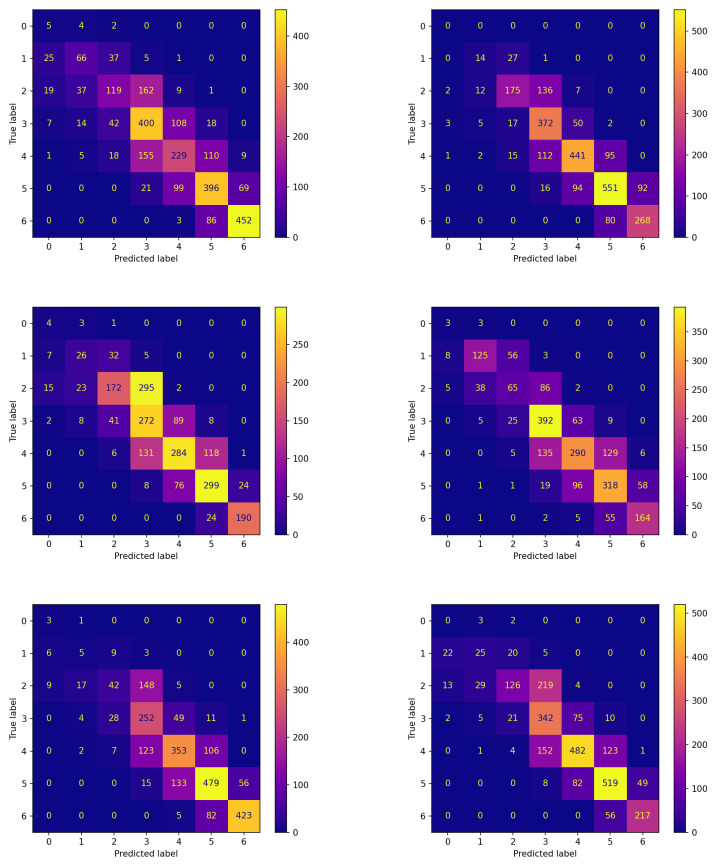
Confusion matrices on the testing set of new subjects not participating in the learning process achieved by the best model (model 570) evolved by non-FL approach.

**Figure 7:**
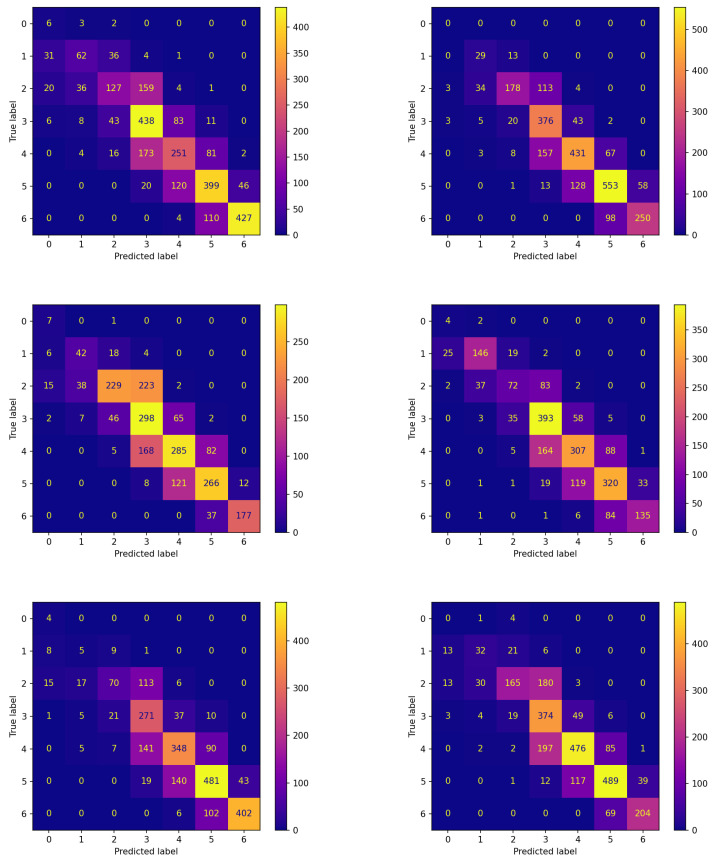
Confusion matrices on the testing set of new subjects not participating in the learning process achieved by the best model (model 575) evolved by FLEA algorithm.

The confusion matrices corresponding to these experiments are reported in Figure 6 and Figure 7 for the two algorithms without and with communication, respectively.

The comparison between the corresponding panes of the two figures evidences that the items are more frequently assigned correctly when FLEA is considered: the cells along the diagonals contain higher values when communication takes place. This is of crucial importance for the two classes corresponding to hypoglycemic events. In fact, the latter are high-risk situations, therefore correctly predicting them well in advance is a major issue for subjects’ health. Moreover, in this case, the results confirm that the model produced by the FLEA algorithm is more general and can be useful when new subjects are to be monitored from scratch.

Table 8 reports a comprehensive view of the six subjects’ numerical scores. This table further confirms that the model achieved by FLEA, on average, performs better than that obtained by the non-FL approach. Specifically, the former shows an improvement of about 3.03% for precision, 1.56% for recall, 3.17% for $F_{1}$, and 1.56% for accuracy.

### 4.3. Statistical Analysis

A statistical analysis test was executed to assess whether or not the best model proposed by the FLEA algorithm performs better than that obtained by the non-FL one over the complete set of the twelve subjects making up the Ohio T1DM data set. This analysis was carried out on the online web platform ‘Statistical Tests for Algorithms Comparison’ [94] (STAC) (https://tec.citius.usc.es/stac/ accessed on 15 January 2023). From among the several statistical tests available, the Quade test was chosen because it considers the higher difficulty of some problems and the larger differences that may be shown by the various algorithms over them; hence, the Quade test is different from the Friedman and Aligned Friedman ones, in which all the problems are considered to be of equal importance. For more details on the statistical analysis shown here in general, and on the Quade test in particular, interested readers can refer to the widely cited paper by [95].

Before running a statistical test, a null hypothesis $H_{0}$ must be chosen; we set it as the fact that the two models proposed by the two algorithms are statistically equivalent. Moreover, a significance level α must be chosen; we set its value as 0.05, which means that, if the null hypothesis is rejected by the test, there is a 5% probability of incorrectly rejecting it.

Table 9 reports the results of this test: the first column contains the algorithms compared, and the second the corresponding rank value; better algorithms are characterized by lower ranking values.

The table shows that FLEA performs better than non-FL on this test. Yet, as the computed *p*-value is 0.16680, which is higher than 0.05, this test cannot exclude the statistical equivalence between the two algorithms.

To further investigate this issue, we must make reference to post hoc procedures, also described in [95]. Table 10 reports the results, in terms of adjusted *p*-values, for the complete set of post hoc procedures available in STAC, i.e., Bonferroni–Dunn, Holm, Hochberg, Finner, and Li. The FLEA algorithm was chosen as the control method because it is the algorithm with the lowest ranking value in the Quade test.

To understand the results in the table, each post hoc procedure returns an adjusted *p*-value for non-FL. For all the procedures, this value is lower than the significance level 0.05. This means that the null hypothesis $H_{0}$ of equivalence is rejected by all of them. Hence, FLEA is statistically better than non-FL.

## 5. Conclusions and Future Work

To suitably deal with the issues of data privacy and interpretability, in this paper, we proposed a distributed framework that constitutes an innovative approach to Federated Learning. The framework consists in a master process and a set of slaves: On each of the latter, a Grammatical Evolution algorithm is run that only learns from local private data and generates explicit models that humans can interpret. At given times, a migration process occurs between the slaves through the master, the result of which is that each slave receives the local best models found by the other slaves. This results in an exchange of knowledge between the different nodes merging locally gained knowledge. This process of knowledge exchange allows obtaining local models that can work effectively over all the local sets of private data, i.e., they can be used as global models.

As data privacy and interpretability are highly relevant to the medical field, we applied this framework to a medical problem, i.e., that of the prediction of future glucose values for T1DM patients. This problem is transformed into a seven-class classification task. To assess the importance of this process of knowledge exchange, the framework was experimentally compared with another that only differs in the fact that knowledge exchange does not take place between the slaves.

The results show the importance of this exchange process to create a set of personalized models, each of which can be used as the global model. Moreover, the model obtained by our federated approach showed higher generalization capability than that achieved by the non-FL approach when the two were applied to the data of subjects who did not participate in the learning process. A statistical analysis evidenced the superiority of the FLEA algorithm.

In our future work, the results and behavior shown by our framework on this specific problem must be further investigated on other data sets from the medical domain in which data privacy and interpretable solutions are hard constraints.

Another important step to take in our future work is to perform an experimental investigation to evaluate if an optimal frequency for information exchange exists that allows improving the results without causing too many risks in terms of security for the whole framework. This analysis can be crucial considering that it is well in the field of dEAs that the numerical quality of the results may depend, even more highly, on communication frequency. This could lead to improvement in the results provided by our approach, which could, in this way, perform much better than the model without communication.

Regarding security, it should be evidenced that, currently, the framework we proposed does not deal with the data security problem during the phases of information exchange. As we exploit a distributed approach to evolve a global model, it is highly appropriate to discuss data transmission security. The proposed approach does not propagate patient data (e.g., his/her measured glucose) but rather a compression of his/her metabolic responses in the form of a best-suited model at the given time. Solely, this does not raise a concern; nonetheless, in connection with the known treatment setup, it may give an opportunity to use such representation to build a targeted attack. To avoid an eavesdropping attack, an encrypted communication link between the master and slave nodes has to be established.

Data transmission security is not a concern of this paper and will be a subject of future work. In our future work, we will have to suitably address this problem to define a complete Federated Learning framework that could help in real-world medical trials where data privacy must be guaranteed.

## Figures and Tables

**Table 1:** Review summary and positioning of our paper in the literature.

Existing ML Systems	Examples Refs.	Strengths	Limitations
Non-FL	[43,44,45,46,47,48,49,50,51,52,53,54,55]	May have good	Problems of data privacy -
	[57,58,59,60,61,62,63,64,65,66,67,68,69,71,72]	Numerical performance	Not usable in many fields
Current-FL	[74,75,76,77]	Solve data	Solutions are not
	[78,79]	Privacy problems	Interpretable
Proposed approach	This paper	Solve data	
		Privacy problems -	
		Solutions are	
		Interpretable	

**Table 2:** Protected functions used in the grammar.

Function	Protected Function
plog(x)	log(1+|x|)
psqrt(x)	$\sqrt{\left|x\right|}$
aq(x,y)	$\frac{x}{\sqrt{1+y^{2}}}$

**Table 3:** The seven classes used for the glucose classification problem.

Class	Class ID	Range (mmol/L)	Range (mg/dL)	Action Required
very low	0	<3.0	<54	immediate action
low	1	[3.0–3.9[	[54–70[	hypoalert and monitor
normal-closing-to-hypo	2	[3.9–5[	[70–90[	‘towards hypo’ warning
normal	3	[5.0–7.8[	[90–140[	none
normal-closing-to-hyper	4	[7.8–10.0[	[140–180[	‘towards hyper’ warning
high	5	[10.0–13.9[	[180–250[	alert and monitor
very high	6	≥13.9	≥250	immediate action

**Table 4:** The number of samples in the training/testing (Tr/Ts) sets for each patient ID and class (C0÷C6).

	*C*0	*C*1	*C*2	*C*3	*C*4	*C*5	*C*6
**ID**	**Tr/Ts**	**Tr/Ts**	**Tr/Ts**	**Tr/Ts**	**Tr/Ts**	**Tr/Ts**	**Tr/Ts**
559	60/31	307/47	1452/231	1453/308	1710/442	348/568	2131/438
563	32/0	270/19	1558/120	2043/321	2712/650	2590/889	1053/337
570	14/0	214/12	730/136	1092/177	1544/262	3325/516	3460/1323
575	215/35	759/102	1631/392	2032/434	2335/505	1815/513	978/355
588	25/0	113/4	837/120	1734/226	3341/615	3920/1014	2046/444
591	110/14	307/128	1199/383	1495/561	2365/474	2358/706	1662/185

**Table 5:** Results of the non-FL approach. The best average $F_{1}$ score is reported in bold.

	Patient						*F*_1_ Score
Best Model	559	563	570	575	588	591	Avg (StdDev)
model 559	0.7006	0.5731	0.6803	0.5878	0.6823	0.5486	0.6288 (0.0604)
model 563	0.6820	0.7072	0.8294	0.5679	0.6975	0.5635	0.6746 (0.0907)
model 570	0.7270	0.7233	0.8269	0.6291	0.7312	0.6061	**0.7073 (0.0729)**
model 575	0.7052	0.6726	0.7657	0.6423	0.6935	0.5866	0.6777 (0.0553)
model 588	0.6466	0.5395	0.7167	0.4996	0.7310	0.5134	0.6078 (0.0947)
model 591	0.6577	0.6913	0.7579	0.5637	0.6761	0.5796	0.6544 (0.0663)
Avg *F*_1_ score	0.6865	0.6511	0.7628	0.5817	0.7019	0.5663	
(StdDev)	(0.0278)	(0.0695)	(0.0540)	(0.0469)	(0.0218)	(0.0297)	

**Table 6:** Results of the FLEA algorithm with a communication frequency equal to 100. The best average $F_{1}$ score is reported in bold.

	Patient						*F*_1_ Score
Best Model	559	563	570	575	588	591	Avg (StdDev)
model 559	0.7304	0.7210	0.8047	0.6337	0.7320	0.6015	0.7039 (0.0675)
model 563	0.7278	0.7141	0.7947	0.6113	0.7193	0.6043	0.6953 (0.0673)
model 570	0.7194	0.7312	0.8314	0.5986	0.7235	0.5952	0.6999 (0.0821)
model 575	0.7234	0.7177	0.8016	0.6472	0.7339	0.6052	**0.7048 (0.0632)**
model 588	0.7260	0.7287	0.8206	0.6230	0.7216	0.6020	0.7037 (0.0730)
model 591	0.7260	0.7214	0.8180	0.6199	0.7291	0.5932	0.7013 (0.0750)
Avg *F*_1_ score	0.7255	0.7223	0.8118	0.6223	0.7266	0.6002	
(StdDev)	(0.0035)	(0.0059)	(0.0126)	(0.0155)	(0.0054)	(0.0045)	

**Table 7:** Results obtained by the two global models over the testing sets of six new subjects.

	Patient						$F_{1}$ Score
Model	540	544	552	567	584	596	Avg (StdDev)
non-FL	0.6075	0.7024	0.5748	0.6193	0.6504	0.6524	0.6345 (0.0402)
FLEA	0.6261	0.7036	0.6077	0.6312	0.6667	0.6681	0.6506 (0.0321)

**Table 8:** Overall numerical scores over the testing sets of six new patients.

	ID	Precision	Recall	$F_{1}$	Accuracy
FLEA	540	0.64	0.63	0.63	0.63
	544	0.72	0.70	0.70	0.70
	552	0.65	0.60	0.61	0.60
	567	0.64	0.63	0.63	0.63
	584	0.69	0.67	0.67	0.67
	596	0.71	0.66	0.67	0.66
Avg		0.68	0.65	0.65	0.65
non-FL	540	0.62	0.61	0.61	0.61
	544	0.72	0.70	0.70	0.70
	552	0.62	0.58	0.57	0.58
	567	0.62	0.62	0.62	0.62
	584	0.67	0.66	0.65	0.66
	596	0.69	0.65	0.65	0.65
Avg		0.66	0.64	0.63	0.64

**Table 9:** Quade ranks test over the twelve subjects of the Ohio T1DM data set.

Rank	Algorithm
FLEA	1.26923
Non-FL	1.73077
Statistic: 2.19188	*p*-value: 0.16680

**Table 10:** Post hoc procedures for Quade ranks test over the twelve subjects of the Ohio T1DM data set. FLEA was chosen as the control method.

	Statistic	Bonferroni-Dunn	Holm	Finner	Hochberg	Li
FLEA vs. non-FL	1.99692	0.04583	0.04583	0.04583	0.04583	0.04583

## Data Availability

The authors do not have permission to share the data.

## Abbreviations

The following abbreviations are used in this manuscript:

CGM	Continuous Glucose Monitoring
DNN	Deep Neural Network
EA	Evolutionary Algorithms
FL	Federated Learning
FLEA	Federated Learning-inspired Evolutionary Algorithm
GDPR	General Data Protection Regulation
GE	Grammatical Evolution
ML	Machine Learning
T1DM	Type 1 Diabetes Mellitus
XAI	Explainable Artificial Intelligence
